# The Value of the Chloride Content of the Cerebro-Spinal Fluid in Diagnosis and Prognosis

**Published:** 1928

**Authors:** Herbert Rogers

**Affiliations:** Assistant Pathologist, Bristol General Hospital; Curator of the Pathological Museum, University of Bristol


### THE VALUE OF THE CHLORIDE CONTENT OF THE CEREBRO-SPINAL FLUID IN DIAGNOSIS AND PROGNOSIS.

BY

HERBERT ROGERS, M.B., CH.B.,

*Assistant Pathologist, Bristol General Hospital ;*
*Curator of the Pathological Museum, University of Bristol.*

---

THIS short paper is based upon examinations of cerebro-spinal fluids withdrawn by lumbar puncture from a series of 22 cases. Its object is to bring to notice the value of the estimation of chlorides in the cerebro-spinal fluid in both the diagnosis and prognosis of various conditions included in the general term "meningitis."

Since 1912, when Mestrezat[1] published his monograph on normal and pathological cerebro-spinal fluids, the value of cytological and chemical investigations has become firmly established. The literature upon this subject is considerable, but references to the chloride estimation in particular are few. During the last few years the most valuable contributions to our knowledge of this subject have been the work of Dr. J. G. Greenfield, at the National Hospital for the Paralysed and Epileptic, and I shall quote freely from these.

Willcox and Lyttle,[2] writing on the chemical composition of the spinal fluid in 1923, came to the conclusion that "the chlorides are likely to vary directly with the acuteness of the infective process, and may bear some constant relation to the disease itself." Greenfield and Carmichael[3] are more emphatic, and state that "the chlorides in cerebro-spinal fluid are so constantly between 0·72 per cent. and 0·75 per cent. that any variation above or below these amounts is always of pathological significance."

The fluids obtained from five of the cases in this series appeared to be normal in all respects, and therefore serve as "controls." The percentages of chlorides in these cases were 0·73, 0·72, 0·74, 0·72 and 0·75 respectively, giving an average of 0·73 per cent. These figures agree with the normal limit laid down by Greenfield in the statement quoted above. A rise in the chloride percentage above 0·75 is said to be indicative of renal inadequacy, but I have no case of this type in which renal efficiency tests were carried out. The majority of the remainder of the cases show a fall in the chloride percentage, which is associated, in a general way, with meningitis.

The cause of the fall in chlorides is still unknown, but most investigators attempt to explain it as being due to a change in the composition of the blood, to a change in the permeability of the choroid plexus, to bacterial activity, or to a combination of these phenomena.

Greenfield's[4] view seems to me to be the most acceptable at the present time, *i.e.* that "this fall in the chlorides is due to a breaking down of the barrier which separates the blood and cerebro-spinal fluid. As Mestrezat termed it, there is an increase of meningeal permeability. In health the chlorides in the cerebro-spinal fluid are always higher than in the blood, but when the barrier between the two fluids is reduced their chloride percentages tend to approximate to one another."

In the consideration of the value of the chloride estimation for diagnostic purposes in meningeal infections it must be remembered that a fall in the chloride percentage in the cerebro-spinal fluid occurs in certain other conditions, such as lobar pneumonia, typhoid and typhus fevers, and in tuberculosis. It is evident, therefore, that this estimation must always be used in conjunction with other investigations of the fluid, for instance, the cytology, protein estimation, globulin, etc. This does not in any way detract from the value of the estimation, but rather enhances it; for it is the modifying influence of a knowledge of the chloride percentage on the picture given by the other investigations that makes it of such importance.

A knowledge of the chloride percentage is of special value in cases of meningitis secondary to inflammatory or suppurative processes elsewhere in the body, particularly in the middle ear, mastoid or other cranial air sinuses. For instance, a case of chronic middle ear suppuration showing clinical signs of meningitis, with increased cells and protein in the cerebro-spinal fluid, may have a widespread meningitis or may not. A chloride percentage within normal limits in such a case would indicate that the meningeal involvement was still localised, and that a timely operation to deal with the source of infection might avert the serious catastrophe of generalised meningitis. Greenfield,[4] reviewing a series of seventeen cases of head injuries, says, in connection with chloride estimations, that "this means that the chlorides always fall when meningitis becomes generalised, and therefore so long as they remain high there is ground for belief that the area of inflammation is localised and for hoping that it may remain so."

Seven cases of this series fall into this group of "secondary meningitis."

The first, with a chloride percentage of 0·585, was a fatal case of widespread meningitis secondary to otitis media.

The second, a boy 9 years of age, had meningitis secondary to mastoiditis. The chloride percentage in the cerebro-spinal fluid was 0·63. Operation relieved the condition and he went out cured.

The third case was one of frontal abscess secondary to sinusitis, with a chloride of 0·685 per cent. and a cell count of 88 polynuclears per c.mm., indicative of an established meningitis.

The fourth case was one of an old cerebellar abscess which began to leak, setting up a generalised meningitis. The cell count was very high, 8,000 polymorphs per c.mm., and a chloride percentage of 0·625. An interesting feature of this case was that a pure culture of Friedlander's pneumobacillus was obtained from the abscess cavity.

The fifth case, presenting signs of cerebellar abscess and having a history of otorrhœa, showed a cell count of 500 per c.mm., polymorphs and lymphocytes in equal proportion, and a chloride percentage of 0·715. At operation an extradural abscess was found accounting for the findings in the cerebro-spinal fluid, but further exploration for the cerebellar abscess was unsuccessful.

The sixth case was admitted to hospital unconscious and hemiplegic. Three months previously the blood had given a triple-positive Wassermann reaction, and a diagnosis of cerebral hemorrhage was favoured. The cerebro-spinal fluid was yellowish in colour, showed increased protein and globulin, and a chloride percentage of 0·70. Post-mortem examination revealed an extradural abscess over the petrous portion of the temporal bone, and a small collection of pus on the surface of the cerebellum.

The seventh case was one of suppurative otitis media ; the cerebro-spinal fluid was greenish in colour, the cell count showed 3,600 polymorphs per c.mm. and a chloride of 0·69 per cent. Operation failed to relieve her, and at post-mortem examination there was extensive purulent meningitis due to pneumococcal infection from the ear.

The fifth and sixth cases appear to me to be examples of fairly localised conditions in which operation is indicated, the deciding feature in each being the relatively high chloride percentage in a cerebro-spinal fluid which is otherwise typical of generalised meningitis. Yerger,[5] writing in 1925 on the intra-cranial complications of nasal and aural conditions, depends entirely upon the cell count of the cerebro-spinal fluid for differentiation between circumscribed and diffuse meningitis. I have not been able to find any satisfactory basis for differentiation by this method; in fact, the cell count can be very misleading.

For example, I have mentioned one case of diffuse meningitis with a cell count of 88 polymorphs, and another case of localised abscess with a count of 500 polymorphs. Differentiation by means of the chloride estimation is more reliable, but tends to be too delicate. The initial involvement of the meninges in these cases may so rapidly pass on to a generalised meningitis that the fall in chlorides may be very sudden, and twenty-four hours will suffice to lower a percentage from normal to about 0·675. For this reason cerebro-spinal fluids from cases of secondary meningitis often show a chloride percentage of about 0·7 or 0·69, and in spite of immediate operation the next sample shows a much lower percentage. But there is at least a chance that the condition will subside after the removal of the cause.

Of the remaining ten cases in this series five are of tuberculous meningitis, four of meningococcal meningitis and one of post-vaccinal encephalitis. In the cases of tuberculous meningitis the chloride percentages were 0·64, 0·628, 0·68, 0·63 and 0·665 respectively. Greenfield regards chloride percentages between 0·6 and 0·5 as pathognomonic of tuberculous meningitis, and indeed it is customary to associate this condition with "lymphocytes and low chlorides."

For this reason there is a tendency to forget that a meningitis in a child, with a cell count chiefly polynuclear and only a moderate reduction of chlorides, may also be of tuberculous origin.

The case which showed a chloride percentage of 0·64 gave a cell count of 43 per cmm. mainly polynuclear, and the case showing a chloride of 0·665 per cent. gave a cell count of 400 with polynuclears and lymphocytes in equal proportion. This last case was also interesting in that the patient was twenty years of age. At no time during her illness could Kernig's sign be elicited. Tubercle bacilli were present in the cerebro-spinal fluid during life, and the diagnosis was also verified post-mortem.

In the four meningococcal meningitis cases the chlorides were 0·615, 0·585, 0·66, and 0·675 per cent. respectively on first examination. The first of these cases improved rapidly under treatment, and the chlorides rose steadily. The second case, showing the initial low chloride percentage of 0·585 improved to 0·64, then relapsed to 0·6; he again improved and chlorides reached 0·705 per cent. Another slight drop occurred to 0·695 per cent., and the case continued in a chronic condition. He eventually left hospital apparently cured. This relapse occurring in meningococcal meningitis at the end of the first week of treatment is of fairly common occurrence, and the reason generally given is that the serum is not reaching the ventricles in sufficient concentration to deal with the infection.

The variations in the chloride estimations which occurred in this case closely followed the actual clinical state of the patient, and I find that a record of the chloride percentages in successive samples of fluid from a given case forms a most reliable index of progress. The last case of meningococcal meningitis remained undiagnosed for some days, owing to failure to isolate the organism, and during that time the chlorides fell from 0·675 to 0·595 per cent. When the exact nature of the infection became evident serum treatment produced a rise in the chloride percentage to 0·665, but the patient died the following day. Caffey and McLean,[6] reviewing thirty cases of meningococcal meningitis, conclude that the chloride estimations of the cerebro-spinal fluid do not give diagnostic or prognostic information of great value, but it seems that they regard the normal range of chlorides as 0·725 to 0·675 per cent., whereas any percentage below 0·70 is considered pathological in this country.

The last case of the series is that of a child admitted to hospital unconscious, ten days after vaccination. The condition was diagnosed as being a post-vaccinal encephalitis. The cerebro-spinal fluid showed 0·2 per cent. total protein, no increase of globulin, a cell count of 16 per cmm. chiefly polynuclears, and a chloride percentage of 0·74. She left hospital apparently cured.

The number of cases of secondary meningitis dealt with in this paper is quite insufficient to give conclusive evidence of the value of the chloride estimation in the differentiation between circumscribed and diffuse meningitis, although they indicate that such a possibility does exist. This estimation is, therefore, worth further investigation, and a greater amount of consideration is due to it than it has received in the past. As an index of progress a record of the chloride percentage in successive samples of fluid from any given case of meningitis is of definite value in prognosis ; the upward curve of percentages encourages a hopeful outlook, while falling percentages are of bad omen. I have shown, however, that the chloride percentage may fall to a very low level, and recovery eventually ensue. From this it follows that in established meningitis no single chloride estimation is of any value in prognosis.

Greenfield,[4] however, says that he has never known a patient die from meningitis with the chlorides at their normal level.

| Case No. | Age. | Clinical Features. | Cerebro-Spinal Fluid. | | | |
|---|---|---|---|---|---|---|
| | | | Cells per c.mm. | Protein per cent. | Globulin. | Chlorides per cent. |
| 1 | — | Normal. | 2 | 0·02 | No increase. | 0·73 |
| 2 | 47 | Normal. | 1 | 0·015 | No increase. | 0·72 |
| 3 | — | Normal. | 3 | 0·015 | No increase. | 0·74 |
| 4 | 30 | Normal. | 2 | 0·015 | No increase. | 0·72 |
| 5 | 47 | Normal. | 3 | 0·015 | No increase. | 0·75 |
| 6 | 39 | Meningitis secondary to otitis media. Confirmed by autopsy. | p. | 0·2++ | Increased. | 0·585 |
| 7 | 9 | Meningitis secondary to mastoiditis. Operation followed by recovery. | 725 p. | 0·15 | Increased. | 0·63 |
| 8 | 15 | Meningitis. Frontal abscess, secondary to sinusitis. Confirmed by autopsy. | 88 p. | 0·025 | No increase. | 0·685 |
| 9 | 16 | Meningitis. Old cerebellar abscess found post-mortem. | 8,000 p. | 0·05 | — | 0·625 |
| 10 | 40 | Meningitis. Extra-dural abscess, secondary to otitis media. Confirmed at autopsy. | 500 equal p. and l. | 0·03 | No increase. | 0·715 |

p = polymorphonuclears. l = lymphocytes

| Case No. | Age. | Clinical Features. | Cerebro-Spinal Fluid. | | | |
|---|---|---|---|---|---|---|
| | | | Cells per c.mm. | Protein per cent. | Globulin. | Chlorides per cent. |
| 11 | 42 | Cerebral hemorrhage suspected during life. Extradural abscess, with localised suppuration on cerebellar surface, found post-mortem. | — | 0·2 | Increased. | 0·7 |
| 12 | 61 | Meningitis secondary to otitis media. Confirmed by autopsy. | 3,500 cells, 75 per cent. p. | 0·2 | Increased. | 0·69 |
| 13 | $1\frac{8}{12}$ | Tuberculous meningitis. Bacilli in C.S.F. Fatal. | 43 p. | 0·045 | Increased. | 0·64 |
| 14 | $4\frac{6}{12}$ | Fatal. No autopsy. | Many l. | — | — | 0·628 |
| 15 | — | Fatal. No autopsy. | 112 l. | 0·045 | — | 0·68 |
| 16 | $1\frac{8}{12}$ | Bacilli in C.S.F. Fatal. | 73·50 per cent. p. | 0·15 | — | 0·63 |
| 17 | 22 | Bacilli in C.S.F. Diagnosis confirmed at autopsy. | 400, p. and l. equal. | 0·1 | Increased. | 0·64 |
| 18 | 6 | Meningococcal meningitis. | Many p. | 0·025 | — | 0·615 |
| 19 | 12 | Meningococcal meningitis. | Many p. | 0·2++ | Increased. | 0·585 |
| 20 | $\frac{6}{12}$ | Meningococcal meningitis. | Many p. | 0·05 | — | 0·66 |
| 21 | 43 | Meningococcal meningitis confirmed at autopsy. | Many p. | 0·05 | No increase. | 0·675 |
| 22 | 7 | Post-vaccinal encephalitis. Recovery. | 16 p. | 0·02 | No increase. | 0·74 |

p=polymorphonuclears. l=lymphocytes.

REFERENCES.

[1] Mestrezat, *Le liquide cephalo-rachidien normel et pathologique*, 1912.

[2] Willcox and Lyttle, *Am. Jour. Dis. Child.*, Chicago, 1925, xxx. 513.

[3] Greenfield and Carmichael, *The Cerebro-Spinal Fluid in Diagnosis.*

[4] Greenfield, *Proc. Roy. Soc. Med.*, 1925, xviii. 9–10. (Sect. Med., Otol. Laryn. Neurol.)

[5] Yerger, *Jour. Am. Med. Assoc.*, 1925, lxxxv 424.

[6] Caffey and McLean, *Jour. Am. Med. Assoc.*, 1927., lxxxviii. 1,859.

